# Predicting the final response and remission of electroconvulsive therapy in treating major depressive disorder: exploring the earliest predictive factors for early improvement

**DOI:** 10.1186/s12888-026-08141-7

**Published:** 2026-05-11

**Authors:** Nanxue Duan, Jie Hu, Jian Guan, Yang Ji, Wanling Huang, Rui Qian, Hao Zheng, Yuhui Wan, Kai Wang, Guixian Xiao, Dai Zhang, Yanghua Tian

**Affiliations:** 1 Department of Neurology, The First Affiliated Hospital of Anhui Medical University, 218 Jixi Road, Shushan District, Hefei, Anhui Province 230022 China; 2 Department of Neurology, The Second Affiliated Hospital of Anhui Medical University, Hefei, 230601 China; 3 Center for Big Data and Population Health of IHM (Anhui Medical University), School of Public Health, Anhui Medical University, Hefei, 230032 China; 4 The College of Mental Health and Psychological Sciences, Anhui Medical University, Hefei, 230032 China; 5 Collaborative Innovation Center of Neuropsychiatric Disorders and Mental Health, Hefei, 230032 China; 6 Anhui Province Key Laboratory of Cognition and Neuropsychiatric Disorders, Hefei, 230022 China; 7 Institute of Artificial Intelligence, Hefei Comprehensive National Science Center, Hefei, 230088 China; 8 Department of Psychology and Sleep Medicine, The Second Affiliated Hospital of Anhui Medical University, Hefei, 230601 China; 9 Department of Radiology, the Second Affiliated Hospital of Anhui Medical University, Hefei, 230601 China

**Keywords:** Major depressive disorder, Electroconvulsive therapy, Early improvement, Predict, Response, Remission

## Abstract

**Background:**

To date, no study has investigated the earliest predictors of early improvement during electroconvulsive therapy (ECT) in patients with major depressive disorder (MDD). The primary objective of this study was to compare the predictive ability of the improvement rate in the 17-item Hamilton Depression Rating Scale (HAMD-17) scores after each ECT session for final treatment response and remission, thereby identifying the earliest early improvement predictors for ECT efficacy in MDD.

**Methods:**

HAMD-17 scores were collected from 143 patients with MDD after each ECT session. Treatment response was defined as a ≥ 50% reduction in HAMD-17 scores from baseline, and remission was defined as a HAMD-17 score ≤ 7. Predictive factors identified as significant in univariate analyses (*p* < 0.05) were further evaluated using receiver operating characteristic (ROC) curve analysis. An area under the ROC curve (AUC) value > 0.8 was considered to indicate good discriminative ability. The earliest time point at which the AUC first exceeded 0.8 was determined as the earliest early improvement predictor for treatment outcomes. The optimal threshold of early improvement for predicting response and remission was determined using the Youden index.

**Results:**

Of the 143 patients who received ECT, 76.92% achieved treatment response and 58.04% achieved remission. An improvement rate of ≥ 42.21% in HAMD-17 scores after three ECT sessions optimally predicted final response, while an improvement rate of ≥ 47.05% after four sessions optimally predicted final remission.

**Conclusion:**

Early improvement during the course of ECT has predictive value for treatment outcomes. Close monitoring of treatment efficacy in the early stages of ECT may facilitate timely adjustment of treatment strategies and improve overall outcomes.

**Supplementary Information:**

The online version contains supplementary material available at 10.1186/s12888-026-08141-7.

## Introduction

Electroconvulsive therapy (ECT) is an important treatment method for patients with severe or treatment-resistant major depressive disorder (MDD), capable of producing rapid and robust antidepressant effects [1]. Its efficacy and safety have been extensively documented [2, 3]. Previous studies have reported pooled response rates of 60%-80% and remission rates of 50%-60% for ECT in MDD [2, 4], indicating that there is significant individual variability in the efficacy of ECT for MDD, and some patients remain unresponsive to ECT treatment. At present, no reliable predictive indicators are available to assess the therapeutic efficacy of ECT.

Numerous studies have explored the relationship between clinical variables and the efficacy of ECT, identifying several factors associated with treatment outcomes. Most evidence suggests that age is a significant predictor, with older patients demonstrating greater treatment efficacy [5–8]. However, some studies have not confirmed an association between older age and improved ECT outcomes [9, 10]. Su et al. (2023) reported that older patients exhibited poorer responses, whereas greater baseline depressive severity was predictive of faster treatment response [11]. Other investigations have indicated that the presence of psychotic symptoms is a stronger predictor of remission than age [12]. Furthermore, evidence indicates a significant negative correlation between ECT response rates and the duration of the depressive episode [13], with better outcomes observed in patients without self-injurious behaviors [14]. Taken together, predictions of ECT efficacy based on these traditional clinical variables remain inconsistent, making it difficult to establish a consensus. Further research is therefore required to identify more reliable clinical predictors of treatment response.

In the study of depressive symptom improvement during ECT, it has been observed that the reduction in core depressive symptoms occurs most rapidly in the early stages of treatment, followed by a gradual deceleration [11]. Some studies have reported that after only three ECT sessions, more than half of patients may show a therapeutic response, which is six times the improvement achieved in subsequent treatment sessions [15]. However, evidence remains limited regarding whether the rate of early improvement can reliably predict final treatment outcomes, and the findings to date are inconsistent. As early as 1998, Gupta et al. (1998) reported that the response to the first ECT session could serve as a feasible predictor of treatment efficacy [16]. Birkenhager et al. (2019) further suggested that a 15% reduction in Montgomery Åsberg Depression Rating Scale (MADRS) scores after two sessions could predict treatment response [17]. Nevertheless, the area under the receiver operating characteristic (ROC) curve (AUC) for this indicator was modest, and its overall predictive accuracy limited. Other investigations have suggested that improvements after three ECT sessions are the most reliable early predictors of ultimate therapeutic efficacy [18, 19]. However, the sample sizes of these studies were small, and further research with larger sample sizes is needed to validate these findings. In contrast, Lin et al. (2016) argued that improvements observed after six sessions provide a better prediction of response and remission [20], a finding supported by some subsequent studies [21, 22]. However, the limitation of using six treatments as an early improvement indicator to predict final treatment efficacy lies in the fact that most depression patients undergo at least six ECT sessions. For some patients, six ECT sessions may be the final treatment, and thus cannot be considered early.

Predicting the ultimate efficacy of ECT during the early stages of treatment is crucial for reducing unnecessary side effects in non-responders and for optimizing clinical decision-making. Previous studies have rarely examined the predictive value of early improvement in relation to ECT efficacy, and no objective comparisons have been made regarding the predictive role of improvement rates after early ECT sessions in determining final outcomes. The present study aims to investigate the predictive value of improvement rates following each ECT session (within the first six sessions) for treatment endpoint response and remission status in patients with MDD. The objective is to identify the earliest indicators of predictive value for early improvement. Additionally, the Youden index was calculated to determine the optimal cut-off value (optimal threshold) for defining the percentage of early improvement. Finally, the predictive ability of clinical variables on treatment endpoint response and remission was also evaluated.

## Methods

### Subjects

This study recruited patients with MDD requiring ECT from inpatients at the Anhui Mental Health Center between February 2023 and October 2024. The inclusion criteria were: meeting the diagnostic criteria for depression according to the Diagnostic and Statistical Manual of Mental Disorders, Fifth Edition (DSM-V), confirmed independently by two psychiatrists; requiring ECT treatment; and being between 14 and 65 years of age. Exclusion criteria included: patients with schizophrenia, schizoaffective disorder, bipolar disorder, or other primary psychotic disorders were excluded; severe physical illnesses; a history of neurological disorders (such as traumatic brain injury or dementia); low educational attainment; or ECT treatment within the previous six months. The severity of depression was assessed using the 17-item Hamilton Depression Rating Scale (HAMD-17) [23] 12–24 h before the first ECT session and 12–24 h after each subsequent session. All assessment timepoints were scored by the same systematically trained psychological assessor, who was not involved in decisions regarding ECT treatment duration. Treatment response was defined as a ≥ 50% reduction in the HAMD-17 total score at the end of treatment, while remission was defined as a HAMD-17 total score of ≤ 7 at the end of treatment [2]. This study was conducted in accordance with the latest revision of the Declaration of Helsinki and was approved by the Ethics Committee of Anhui Medical University (approval number: 84230093). Written informed consent was obtained from the parents, guardians, or close relatives of all minor participants. All adult participants provided written informed consent prior to enrolment.

### ECT procedures

According to the Chinese Expert Consensus on Modified Electroconvulsive Therapy (2019 Edition) guidelines, ECT was administered at the Anhui Mental Health Center using the Thymatron System IV Integrated ECT System (Somatics, Lake Bluff, IL, USA). The number of ECT sessions ranged from 6 to 10. The first three sessions were delivered on consecutive days, followed by sessions every other day, with weekends excluded. All patients received propofol (1.4 mg/kg), succinylcholine (0.5 mg/kg), and atropine (0.5 mg) prior to each ECT session, for the induction of anesthesia, muscle relaxation, and inhibition of glandular secretion, respectively. Electroconvulsive stimulation was then administered with the following parameters: bifrontal electrode placement, 0.9 A constant current, 0.5 ms pulse width. The energy percentage for the first ECT session was set according to patient age (e.g., 50% for a 50-year-old patient, approximately 250 mC). If the seizure was inadequate, the stimulation energy percentage was increased by 5% (approximately 25 mC) in the subsequent session. No restimulation was administered within the same session if the initial stimulation failed. This titration process continued across sessions until an adequate seizure was achieved [24, 25]. Seizure activity was continuously monitored using electroencephalography during the procedure, with the minimum seizure duration defined as 25 s [26]. Following each ECT session, patients were transferred to a recovery room for continuous monitoring of vital signs until full recovery of consciousness and stabilization of vital parameters were confirmed prior to discharge. Concomitant pharmacological treatments were maintained unchanged throughout the ECT course, with detailed medication information provided in Table 1.

**Table 1:** Demographic information and clinical characteristics

	Total	Response ^a^				Remission ^b^			
		Responders	Non-responders	Statistics	*p*-value	Remitters	Non-remitters	Statistics	*p*-value
Sample size	143	110	33	-	-	83	60	-	-
Age (year)	33.02 ± 13.03	34.66 ± 12.80	27.55 ± 12.48	*U* = 1201.500	0.003 ^c^	35.14 ± 12.45	30.08 ± 13.35	*U* = 1843.500	0.008 ^c^
Sex (male/female)	55/88	42/68	13/20	*χ*^*2*^ = 0.016	0.900 ^d^	34/49	21/39	*χ*^*2*^ = 0.523	0.469 ^d^
Education level (years)	11.05 ± 3.78	10.85 ± 4.03	11.70 ± 2.73	*U* = 1616.500	0.337 ^c^	10.93 ± 4.00	11.22 ± 3.47	*U* = 2346.000	0.552 ^c^
Episodes (first/recurrence)	34/109	28/82	6/27	*χ*^*2*^ = 0.741	0.389 ^d^	22/61	12/48	*χ*^*2*^ = 0.813	0.367 ^d^
Duration of illness (month)	73.88 ± 73.26	78.30 ± 78.02	59.15 ± 46.33	*U* = 1695.500	0.566 ^c^	77.02 ± 78.66	69.53 ± 62.73	*U* = 2468.500	0.930 ^c^
Duration of current episodes (month)	5.85 ± 7.73	5.72 ± 6.97	6.31 ± 9.97	*U* = 1780.000	0.866 ^c^	5.68 ± 6.55	6.09 ± 9.16	*U* = 2311.000	0.461 ^c^
Participants with suicide attempts (yes/no)	29/114	20/90	9/24	*χ*^*2*^ = 1.298	0.255 ^d^	14/69	15/45	*χ*^*2*^ = 1.425	0.233 ^d^
Participants with Psychotic symptoms (yes/no)	25/118	18/92	7/26	*χ*^*2*^ = 0.414	0.520 ^d^	13/70	12/48	*χ*^*2*^ = 0.454	0.500 ^d^
Family history of mental illness (yes/no)	15/128	11/99	4/29	*-*	0.727 ^e^	9/74	6/54	*χ*^*2*^ = 0.026	0.871 ^d^
Number of ECT sessions	7.88 ± 1.14	7.90 ± 1.14	7.82 ± 1.16	*U* = 1747.000	0.704 ^c^	7.93 ± 1.15	7.80 ± 1.13	*U* = 2370.000	0.567 ^c^
Baseline HAMD score	24.50 ± 6.43	24.43 ± 6.40	24.76 ± 6.61	*t* = -0.258	0.797 ^f^	23.49 ± 6.52	25.90 ± 6.08	*t* = -2.240	0.027 ^f^
Improvement rate in total HAMD after the first ECT (%)	23.76 ± 20.44	25.25 ± 21.19	18.78 ± 17.10	*U* = 1399.500	0.046 ^c^	24.94 ± 22.21	22.12 ± 17.76	*U* = 2218.000	0.266 ^c^
Improvement rate in total HAMD after the second ECT (%)	39.37 ± 23.74	43.26 ± 23.23	26.38 ± 20.89	*U* = 1014.500	<0.001 ^c^	43.94 ± 24.40	33.04 ± 21.43	*t* = 2.772	0.006 ^f^
Improvement rate in total HAMD after the third ECT (%)	47.87 ± 25.41	54.56 ± 22.48	25.59 ± 21.86	*t* = 6.532	<0.001 ^f^	58.65 ± 21.24	32.97 ± 23.19	*t* = 6.864	<0.001 ^f^
Improvement rate in total HAMD after the fourth ECT (%)	52.85 ± 28.20	60.72 ± 24.25	26.61 ± 24.55	*U* = 609.500	<0.001 ^c^	66.07 ± 22.26	34.55 ± 25.28	*U* = 891.000	<0.001 ^c^
Improvement rate in total HAMD after the fifth ECT (%)	58.13 ± 28.41	67.45 ± 22.28	27.04 ± 24.42	*U* = 379.500	<0.001 ^c^	72.80 ± 21.84	37.83 ± 23.63	*U* = 567.000	<0.001 ^c^
Improvement rate in total HAMD after the sixth ECT (%)	65.04 ± 25.95	74.16 ± 18.35	34.63 ± 24.54	*U* = 260.000	<0.001 ^c^	79.95 ± 15.75	44.41 ± 23.02	*U* = 391.000	<0.001 ^c^
Medicine category									
SSRIs (yes/no)	62/81	46/64	16/17	*χ*^*2*^ = 0.459	0.498 ^d^	32/51	30/30	*χ*^*2*^ = 1.858	0.173 ^d^
SNRIs (yes/no)	71/72	56/54	15/18	*χ*^*2*^ = 0.302	0.583 ^d^	45/38	26/34	*χ*^*2*^ = 1.650	0.199 ^d^
SARIs (yes/no)	7/136	5/105	2/31	*-*	0.662 ^e^	5/78	2/58	*-*	0.699 ^e^
NaSSAs (yes/no)	15/128	13/97	2/31	*-*	0.521 ^e^	10/73	5/55	*χ*^*2*^ = 0.512	0.474 ^d^
Antipsychotics (yes/no)	128/15	98/12	30/3	*-*	1.000 ^e^	71/12	57/3	*χ*^*2*^ = 3.318	0.069 ^d^
Anticonvulsants (yes/no)	38/105	31/79	7/26	*χ*^*2*^ = 0.632	0.427 ^d^	18/65	20/40	*χ*^*2*^ = 2.421	0.120 ^d^
Anti-anxiety (yes/no)	25/118	18/92	7/26	*χ*^*2*^ = 0.414	0.520 ^d^	11/72	14/46	*χ*^*2*^ = 2.453	0.117 ^d^
Non-benzodiazepine hypnotic (yes/no)	66/77	53/57	13/20	*χ*^*2*^ = 0.789	0.374 ^d^	40/43	26/34	*χ*^*2*^ = 0.331	0.565 ^d^

Note

^a^ Response: 50% or more reduction in HAMD score at treatment endpoints

^b^ Remission: HAMD score less than or equal to 7 at treatment endpoints

^c^ Mann-Whitney *U* Test

^d^ Pearson’s *χ²* test

^e^ Fisher’s exact test

^f^ Independent *t* test. Abbreviations: ECT, electroconvulsive therapy; HAMD, 17-item Hamilton Depression Rating Scale

### Statistical analysis

Data were analyzed using IBM SPSS Statistics (version 25.0; IBM SPSS Inc., Chicago, IL, USA). Statistical analyses were conducted on patients who completed at least six ECT sessions. Following treatment, participants were categorized into responder/non-responder and remission/non-remission groups according to established response and remission criteria. Demographic and clinical variables were compared between groups, including age, sex, education level, episode type (first episode or recurrent), duration of illness (time from first depressive episode to current admission), duration of current episode, presence of self-injurious behavior, suicide attempts, psychotic symptoms (i.e., delusions or hallucinations), family history of mental illness, number of ECT sessions, baseline depression severity (HAMD-17 score), improvement rate of HAMD-17 score after each ECT session (within the first six sessions), and the use of antidepressant medications. Categorical variables were compared using Pearson’s chi-squared test or Fisher’s exact test, while continuous variables were analyzed using independent samples *t*-test or Mann-Whitney *U* test. A two-tailed *p* < 0.05 was considered statistically significant. Variables identified as significant predictors (*p* < 0.05) in univariate analysis were subsequently entered into ROC analyses. ROC curves were used to determine the optimal predictive factors for predicting final response or remission. The area AUC was used to quantify the test’s ability or accuracy in identifying responder versus non-responder, or remission versus non-remission, with AUC > 0.80 considered indicative of good discrimination [27]. Given that the primary aim was to evaluate the predictive value of early improvement, the earliest ECT session with an improvement rate > 0.80 was defined as “early”. The Youden index was calculated to identify the optimal cut-off value, representing the most accurate definition of “early improvement”. Sensitivity (true positive rate: the proportion of responders/remitters achieving early improvement), specificity (true negative rate: the proportion of non-responders/non-remitters not achieving early improvement), false positive rate (100% - specificity), false negative rate (100% － sensitivity), negative predictive value (NPV: the proportion of patients without early improvement who did not achieve response/remission), and positive predictive value (PPV: the proportion of patients with early improvement who achieved response/remission) were also calculated. Finally, to assess the robustness and generalization capability of the predictive indicators, analysis was performed using R (version 4.5.1) with internal validation via 10-fold cross-validation.

## Results

### Demographic and clinical characteristics

**Fig. 1:**
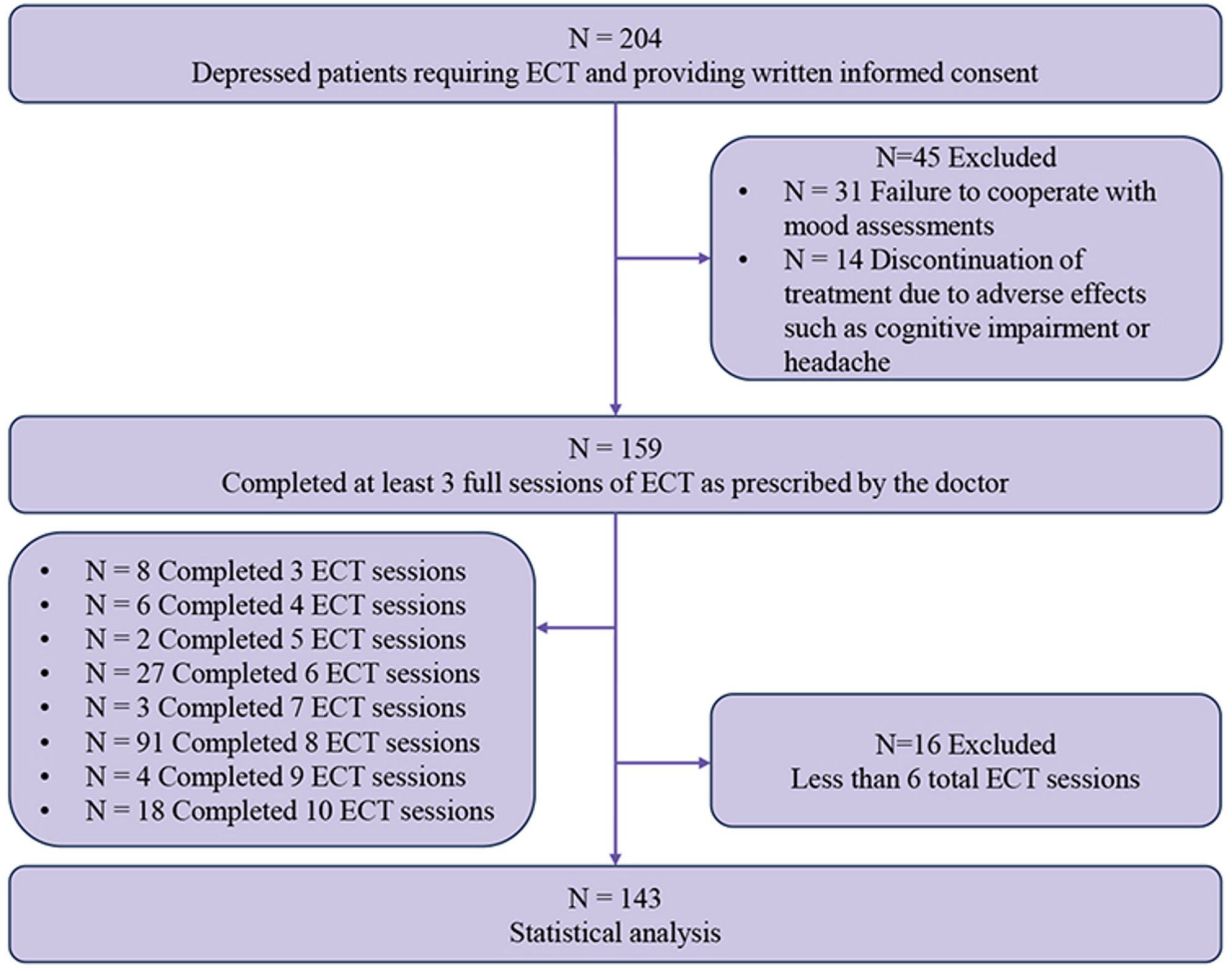
Selection of participants for the analysis. Abbreviations: ECT, electroconvulsive therapy

**Fig. 2:**
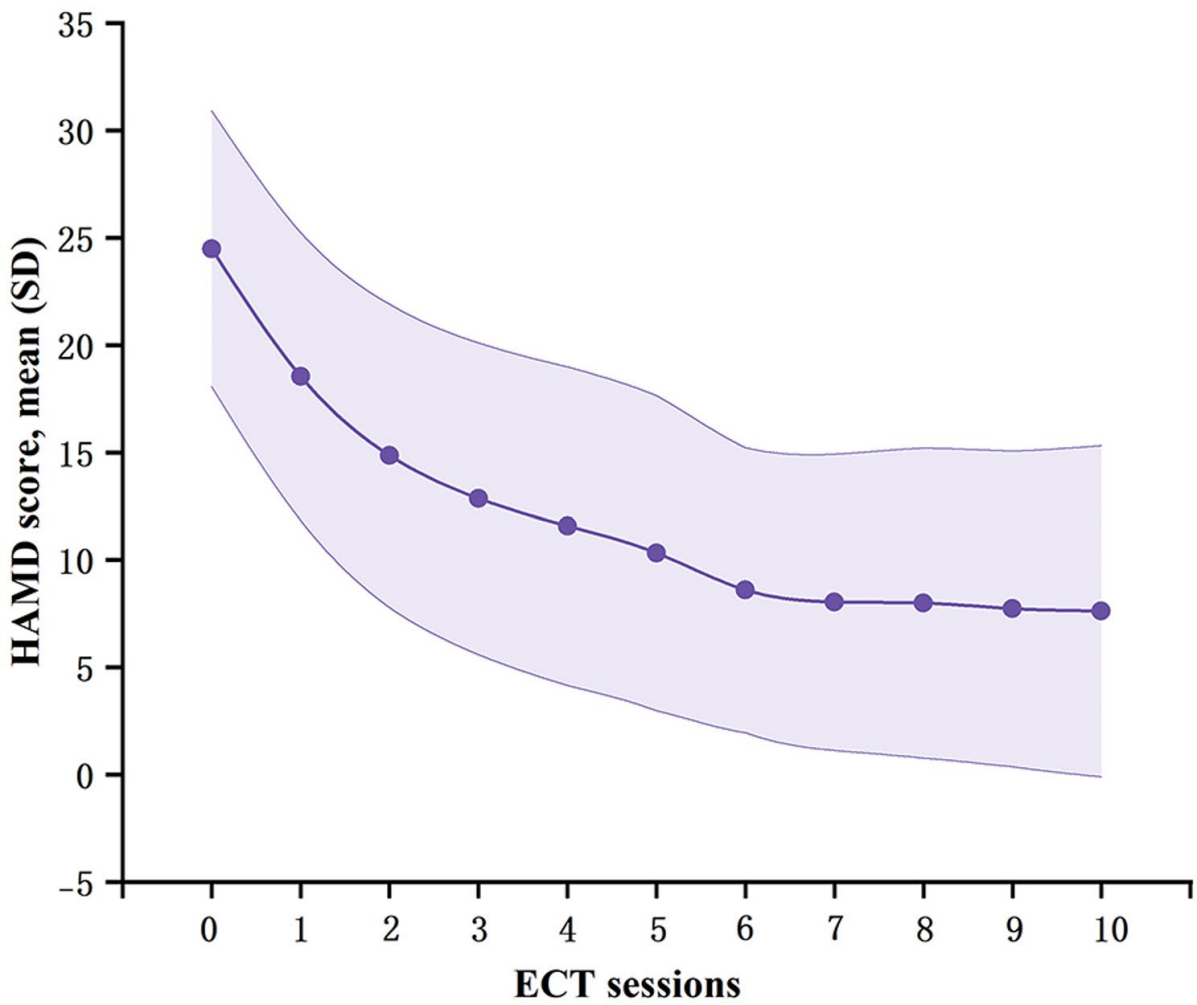
Changes in the HAMD-17 total score over time during ECT. Abbreviations: HAMD-17: 17-item Hamilton Depression Rating Scale; ECT, electroconvulsive therapy; SD, standard deviations

A total of 204 patients with MDD were initially screened for enrollment. Of these, 31 failed to complete the questionnaire assessments, 14 withdrew due to adverse effects of ECT, and 16 were excluded for receiving fewer than six ECT sessions. Ultimately, 143 patients with MDD were included in the final analysis (see Fig. 1 for details). The mean age of the participants was 33.02 years (standard deviation (SD) = 13.03), and 61.54% were female. The mean educational level was 11.05 years (SD = 3.78). Recurrent episodes were present in 76.22% of patients, with a mean illness duration of 73.88 months (SD = 73.26), and the mean duration of the current episode was 5.85 months (SD = 7.73). In total, 20.28% of participants had a history of suicide attempts, 17.48% exhibited psychotic symptoms (such as auditory hallucinations and delusion of persecution), and 10.49% reported a family history of mental illness. On average, patients received 7.88 ECT sessions (SD = 1.14). The mean HAMD-17 score at baseline was 24.50 (SD = 6.43), which significantly decreased to 7.69 (SD = 6.94) after the final ECT session (paired-samples *t*-test, *t* = 25.15, *p* < 0.001). The longitudinal change in HAMD-17 total scores across ECT sessions is illustrated in Fig. 2. Demographic and clinical characteristics, as well as concurrent medication use during ECT, are summarized in Table 1.

### Response and remission

Among the 143 patients with MDD who received ECT treatment, 76.92% (*n* = 110) were classified as responders, and 58.04% (*n* = 83) were classified as remitters at the end of treatment. As shown in Table 1, there were no significant differences between responders and non-responders in sex, education level, episode type (first episode or recurrence), duration of illness, duration of the current episode, presence of suicide attempts, presence of psychotic symptoms, family history of mental illness, number of ECT sessions, baseline HAMD-17 score, or concomitant use of antidepressant medications. However, responders were significantly older (*p* = 0.003) and showed greater improvement rates in HAMD-17 scores after the first (*p* = 0.046), second (*p* < 0.001), third (*p* < 0.001), fourth (*p* < 0.001), fifth (*p* < 0.001), and sixth (*p* < 0.001) ECT sessions compared with non-responders. Similarly, there were no significant differences between remitters and non-remitters in sex, educational level, episode type, duration of illness, duration of the current episode, presence of suicide attempts, presence of psychotic symptoms, family history of mental illness, number of ECT sessions, improvement rate in HAMD-17 scores after the first ECT treatment, and use of antidepressant medications. In contrast, remitters were significantly older (*p* = 0.008), had lower baseline HAMD-17 scores (*p* = 0.027), and exhibited higher improvement rates in HAMD-17 scores after the second (*p* < 0.001), third (*p* < 0.001), fourth (*p* < 0.001), fifth (*p* < 0.001), and sixth (*p* < 0.001) ECT sessions compared with non-remitters.

### Area under the ROC curve (AUC)

**Fig. 3:**
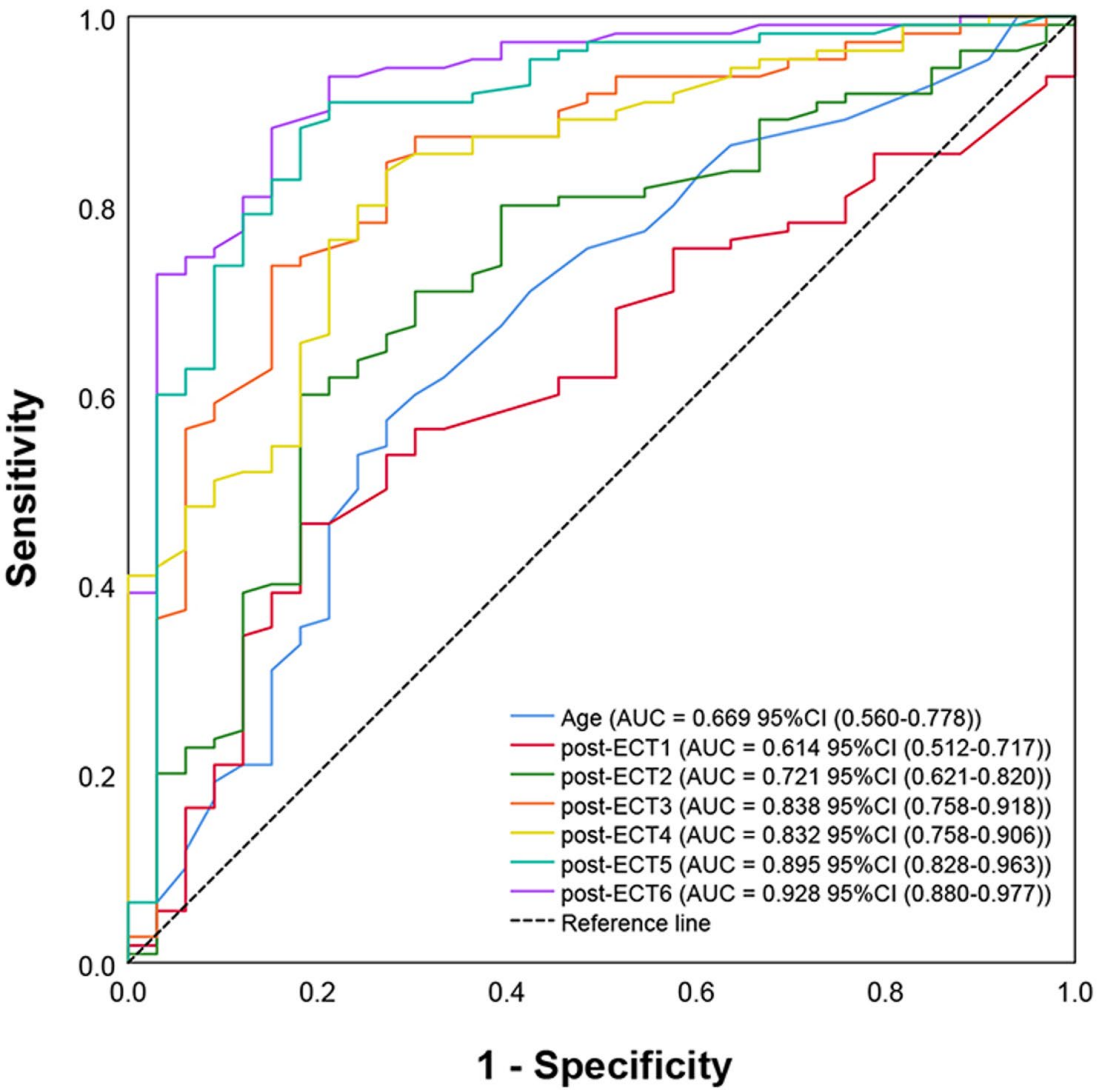
ROC curve analysis is used to predict the final response status of ECT for MDD. Abbreviations: ROC: receiver operating characteristic; ECT, electroconvulsive therapy; MDD, major depressive disorder; AUC, area under the ROC curve; CI, confidence intervals

**Fig. 4:**
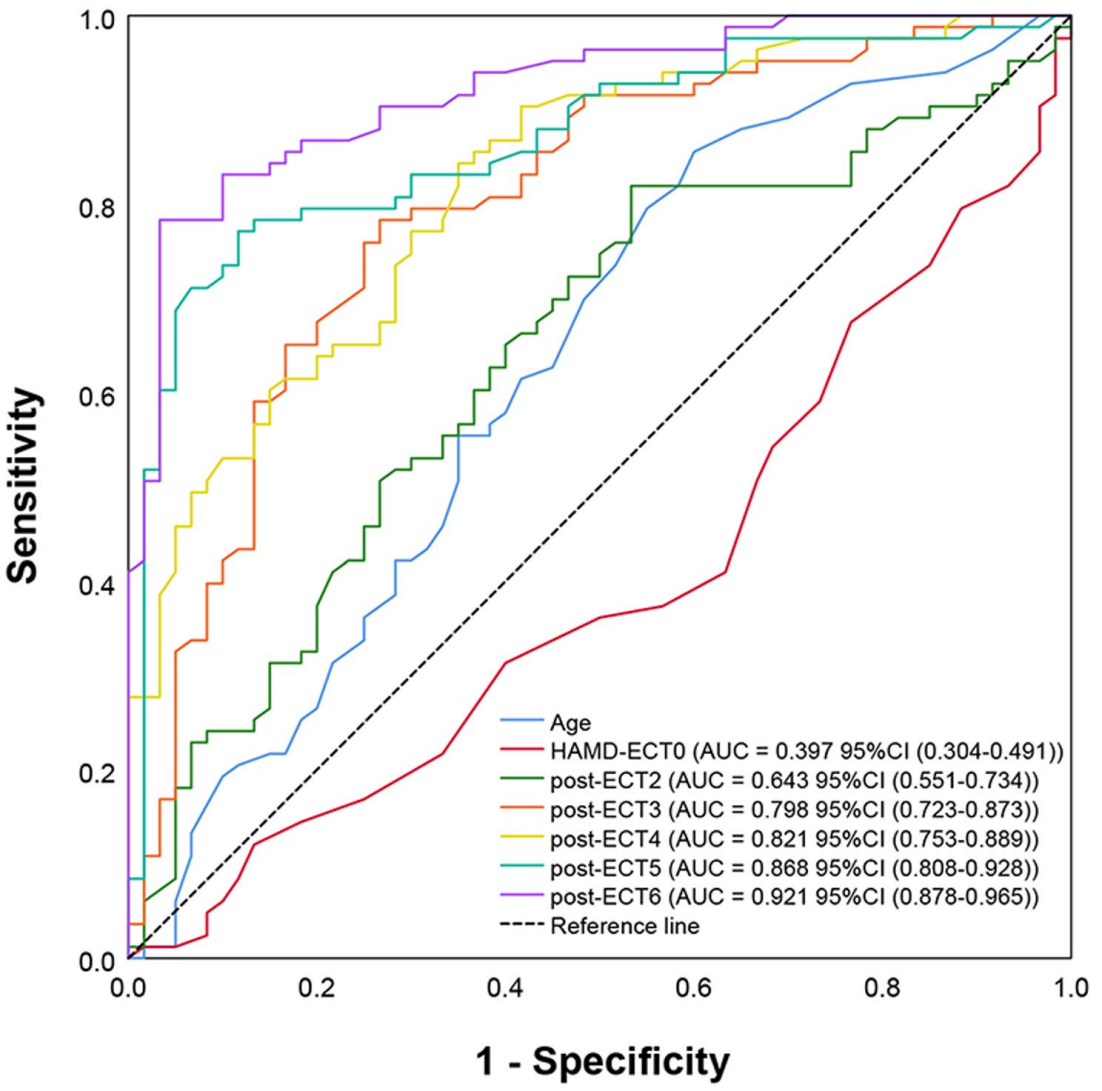
ROC curve analysis is used to predict the final remission status of ECT for MDD. Abbreviations: ROC: receiver operating characteristic; ECT, electroconvulsive therapy; MDD, major depressive disorder; AUC, area under the ROC curve; CI, confidence intervals

As shown in Fig. 3, variables that significantly differed between responders and non-responders—namely age and the improvement rates in HAMD-17 scores after the first through sixth ECT sessions—were entered into ROC curve analyses to identify predictors of final treatment response. Since an AUC value greater than 0.80 indicates good discriminative ability, the third ECT session (AUC = 0.838) was identified as the earliest time point that could reliably predict final response. Similarly, as shown in Fig. 4, variables that significantly differed between remitters and non-remitters, including age, baseline HAMD-17 scores, and improvement rates after the second through sixth ECT sessions, were included in ROC analyses to determine predictors of final remission. The fourth ECT session, at which the AUC value first exceeded 0.80 (AUC = 0.821), was identified as the earliest effective predictor of final remission. Detailed AUC values, SD, and 95% confidence intervals (CI) for each variable are presented in Table 2. All subsequent analyses were therefore based on early improvement after the third ECT session for predicting final response and early improvement after the fourth ECT session predicting final remission.

**Table 2:** ROC analysis of clinical variables for predicting treatment response or remission

Outcome	Clinical variables	AUC (%)	Standard error (%)	95% CI for AUC (%)
Response	Age (year)	66.9	5.5	56.0-77.8
	Improvement rate in total HAMD after the first ECT	61.4	5.2	51.2–71.7
	Improvement rate in total HAMD after the second ECT	72.1	5.1	62.1–82.0
	Improvement rate in total HAMD after the third ECT	83.8	4.1	75.8–91.8
	Improvement rate in total HAMD after the fourth ECT	83.2	3.8	75.8–90.6
	Improvement rate in total HAMD after the fifth ECT	89.5	3.4	82.8–96.3
	Improvement rate in total HAMD after the sixth ECT	92.8	2.5	88.0-97.7
Remission	Age	63.0	4.8	53.5–72.5
	Baseline HAMD score	39.7	4.8	30.4–49.1
	Improvement rate in total HAMD after the second ECT	64.3	4.7	55.1–73.4
	Improvement rate in total HAMD after the third ECT	79.8	3.8	72.3–87.3
	Improvement rate in total HAMD after the fourth ECT	82.1	3.4	75.3–88.9
	Improvement rate in total HAMD after the fifth ECT	86.8	3.0	80.8–92.8
	Improvement rate in total HAMD after the sixth ECT	92.1	2.2	87.8–96.5

Abbreviations: ROC, receiver operating characteristic; AUC, area under the receiver operating characteristic curve; CI, confidence intervals; HAMD, 17-item Hamilton Depression Rating Scale; ECT, electroconvulsive therapy

### Predictive value of early improvement

In the responders and non-responders, ROC curve analysis was performed to predict final treatment response based on the improvement rate in HAMD-17 scores after three ECT sessions. The Youden index was calculated, and a cut-off value of 42.21%, corresponding to the maximum Youden index (0.58), was identified as the optimal threshold. Accordingly, an improvement rate of ≥ 42.21% in HAMD-17 scores after three ECT sessions was considered predictive of a final treatment response. The sensitivity was 73.64%, specificity 84.85%, false positive rate 15.15%, false negative rate 26.36%, PPV 94.19%, NPV 49.12%, and overall accuracy 76.22%. In the remission and non-remission groups, ROC curve analysis for predicting final remission based on the improvement rate of HAMD-17 scores after four ECT sessions identified an optimal cut-off value of 47.05%, determined using the same method. Thus, an improvement rate of ≥ 47.05% in HAMD-17 scores after four ECT sessions was considered predictive of final remission, with a sensitivity of 84.34%, specificity of 65.00%, false positive rate of 35.00%, false negative rate of 15.66%, PPV of 76.92%, NPV of 75.00%, and overall accuracy of 76.22% (Table 3).

**Table 3:** Predictive performance of early HAMD-17 improvement

Outcome	Optimal cut-off values for improvement	Sensitivity (true positive)	Specificity (true negative)	False positive (100% -specificity)	False negative (100% -sensitivity)	PPV	NPV
Response	42.21%	73.64%	84.85%	15.15%	26.36%	94.19%	49.12%
Remission	47.05%	84.34%	65.00%	35.00%	15.66%	76.92%	75.00%

Abbreviations: PPV, positive predictive value; NPV, negative predictive value; HAMD-17, 17-item Hamilton Depression Rating Scale; ECT, electroconvulsive therapy

### 10-fold cross-validation

To validate the robustness of predictive indicators, 10-fold cross-validation was conducted. As shown in Table S1, the AUC value for predicting final response based on the improvement rate of HAMD-17 scores after three ECT sessions was 0.846 (95% CI: 0.744–0.949). with an optimal cut-off value of 38.50%. That is, an improvement rate ≥ 38.50% in the HAMD-17 score after three ECT sessions can predict a final response. The sensitivity of this indicator was 75.52% (95% CI: 66.82%-84.21%), with a specificity of 69.20% (95% CI: 47.87%-90.65%). The AUC for predicting final remission based on the improvement rate of the HAMD-17 score after four ECT sessions was 0.822 (95% CI: 0.703–0.940), with an optimal cut-off value of 45.71%. That is, an improvement rate ≥ 45.71% in the HAMD-17 score after four ECT sessions predicted ultimate remission, with a sensitivity of 84.30% (95% CI: 74.93%-93.71%) with a specificity of 60.00% (95% CI: 38.13%-81.97%). 10-fold cross-validation results indicated that the AUC value for early improvement rate showed no significant change after internal validation, confirming its predictive efficacy as reliable and robust without evident overfitting. Additionally, 10-fold cross-validation provided optimized cut-off values (≥ 38.50% after three treatment sessions, ≥ 45.71% after four sessions). The cut-off after four sessions (45.71%) remained close to the original optimal cut-off (47.05%). However, the cut-off value after three treatment sessions (38.50%) showed a slight decrease compared to the original optimal cut-off (42.21%), yet sensitivity was significantly enhanced.

### Sensitivity analysis

To mitigate bias from dropout data, we conducted a sensitivity analysis to assess the robustness of our findings. Using modified intention-to-treat (mITT) approach, we included patients who completed at least three ECT sessions and had at least two post-baseline assessments (*n* = 159). Within this mITT population, the ECT response rate was 75.47% (120/159), and the remission rate was 54.09% (86/159) (Table S2). The optimal early improvement thresholds for predicting ultimate response and remission were ≥ 42.21% improvement in HAMD-17 scores after three ECT sessions and ≥ 47.06% improvement after four ECT sessions, respectively. For response prediction, the PPV was 94.68% and the NPV was 52.31%. for remission prediction, the PPV was 74.23% and NPV was 75.93% (Table S3 and Table S4). These results closely mirrored the thresholds and predictive values observed in the primary analysis, which included only patients who completed at least six ECT sessions (*n* = 143). This indicates that despite a slight decrease in overall response and remission rates under the more conservative mITT framework, the discriminative performance of early symptom improvement as a predictive indicator, as well as its optimal operational thresholds, remained largely unchanged. Collectively, these findings further support the robustness and clinical applicability of the predictive criteria proposed in this study.

## Discussion

This study aimed to identify the earliest indicators of early improvement that can predict the efficacy of ECT. The findings revealed that (1) 76.92% of patients with MDD achieved a treatment response after ECT, and 58.04% achieved remission, which aligns with recent clinical research findings [28]. (2) An improvement of ≥ 42.21% in HAMD-17 scores after three ECT sessions predicted a final treatment response, while an improvement of ≥ 47.05% after four sessions predicted final remission. (3) Demographic factors such as age, duration of illness, and the presence of psychotic symptoms were not significant predictors of treatment outcomes.

There are few reports on the predictive value of early improvement after ECT for final treatment outcomes. To our knowledge, this study is the first to compare the predictive value of the improvement rate in the HAMD-17 score after each of the first six ECT sessions for predicting final treatment response or remission. An AUC value greater than 0.8 was considered to indicate good discriminatory power, thereby defining the earliest ECT session at which early improvement occurs. Early improvement after three ECT sessions predicted the final treatment response, whereas improvement after four sessions predicted final remission. These findings are consistent with previous research [18], which reported that a 45% reduction in HAMD-17 scores after three ECT sessions could predict final remission. However, in the present study, the improvement rate after three ECT sessions showed limited ability to distinguish final remission (AUC < 0.8), whereas four sessions demonstrated better discriminatory performance. A possible explanation for this discrepancy is the difference in anesthetic regimens: the previous study used a combination of ketamine and propofol (ketofol), whereas propofol alone was used in the current study. Compared with propofol monotherapy, ketofol anesthesia has been shown to significantly enhance the antidepressant efficacy of ECT [29, 30]. Similarly, Çiftçi et al. (2016) identified three ECT sessions as the threshold for early improvement [31], demonstrating that a 21% reduction in HAMD-17 scores after three ECT sessions (one week after treatment initiation) could predict final response, with acceptable specificity (86.67%) but only moderate sensitivity (60.61%). The discrepancy with our results may be attributed to racial or ethnic differences. Notably, the definition of early improvement varies across studies. Some investigations have defined early improvement as a ≥ 20% reduction in HAMD-17 scores after six ECT sessions [20, 22]. However, given that the total ECT course in those studies did not exceed 12 sessions, six sessions more likely represent mid- or late-phase improvement rather than early improvement. Therefore, six sessions may not constitute an optimal time point for defining early improvement [17]. The early improvement indicators identified in this study provide a potential basis for real-time evaluation during the course of ECT treatment. If a patient reaches or exceeds the improvement rate threshold recommended in this study after the third or fourth treatment session, this constitutes a strong positive prognostic signal. Clinicians may use this information to engage in proactive communication with patients and their families, reinforce confidence in the treatment, and proceed with greater assurance in completing the planned treatment course as originally designed. Conversely, failure to achieve the expected early improvement should be regarded as an important “warning signal,” suggesting that the patient’s response to the current treatment parameter regimen may be suboptimal. Importantly, the absence of significant early improvement does not imply that ECT will ultimately be ineffective. Some patients may exhibit a delayed response or remission later in the treatment course; therefore, early non-response alone does not mandate an immediate change in treatment strategy. Instead, a systematic review should first be undertaken to identify potential factors affecting treatment efficacy, such as insufficient seizure quality, the influence of concomitant medications, or underlying medical comorbidities. After these factors have been carefully evaluated and excluded, this warning signal may serve as a basis for more proactive discussions regarding potential adjustments to the treatment strategy, including modification of electrode placement, enhancement of stimulation parameters, or extension of the ECT treatment session.

This study not only used ROC analysis to determine that the improvement rate of the HAMD-17 score after three ECT sessions predicts final treatment response, and that the improvement rate after four sessions predicts final remission, but also evaluated the robustness of these predictive indicators using 10-fold cross-validation. The cross-validation results showed that the AUC values of both predictive indicators exhibited no substantial changes compared with those obtained from the ROC analysis, indicating that both indicators have good generalizability and are not easily affected by sampling variability. However, the optimal cut-off value for the improvement rate of the HAMD-17 score after three ECT sessions re-optimized during cross-validation (38.50%) was slightly lower than the original cut-off value (42.21%) and was associated with higher sensitivity. A possible explanation for this difference is that the original cut-off value was derived from the full sample, whereas optimization of the cut-off value in relatively smaller training sets during cross-validation may favor a slightly lower threshold to improve sensitivity. From a clinical practice perspective, this finding provides important guidance for application. The original cut-off value (42.21%) provides good sensitivity (73.64%) while maintaining relatively high specificity (84.85%), making it suitable for clinical scenarios in which avoiding unnecessary treatment interventions is a priority. In contrast, the optimized cut-off value (38.50%) yields higher sensitivity (75.52%) and may be more appropriate in clinical settings where maximizing the identification of potential responders and minimizing missed diagnoses is required.

It is worth noting that the present study found, in univariate analyses, that the mean age of responders was significantly higher than that of non-responders (*p* < 0.003), and the mean age of remitters was also significantly higher than that of non-remitters (*p* < 0.008), suggesting that older patients with MDD may respond better to ECT. This finding aligns with previous studies [32, 33]. Most studies have reported age as a significant predictor of treatment outcomes [8, 34], indicating that advanced age is associated with more favorable responses to ECT. One possible explanation is that older patients may exhibit reduced tolerance or inadequate response to antidepressant medications, rendering earlier initiation of ECT advantageous for shortening depressive episodes and potentially improving prognosis. In addition, baseline depression severity differed significantly between the remission and non-remission groups (*p* = 0.027), suggesting that patients with more severe baseline symptoms are less likely to achieve remission. This finding is in line with prior studies that have identified baseline depression severity as an important predictor of treatment outcome [35], indicating that poorer outcomes are often associated with greater symptom severity at baseline. In summary, both age and baseline depression severity are important traditional indicators for predicting the efficacy of ECT. The findings of this study support their reference value in comprehensive clinical decision-making. However, when both factors were incorporated into ROC analysis, their predictive ability for ultimate response or remission was found to be limited. Despite this, they retain clinically relevant reference value. Combining them with the improvement rate of early depressive symptoms may further enhance the predictive value for ECT efficacy.

This study aimed to identify early improvement indicators that can predict the outcomes of ECT at the earliest possible stage. Nevertheless, several limitations should be acknowledged. First, the total sample size of 143 participants was relatively small, and future studies with larger cohorts are warranted to validate the present findings. Second, all participants continued taking antidepressant medications during ECT, and thus the potential influence of pharmacotherapy on treatment outcomes cannot be completely excluded. The impact of concurrent antidepressant use on the efficacy of ECT remains a matter of debate [36, 37]. However, in this study, there were no significant differences in antidepressant use between responders and non-responders, or between remitters and non-remitters. The applicability of the present findings to patient populations not receiving antidepressant medications therefore requires further confirmation. Third, this study employed only one evaluator, precluding the calculation of inter-rater reliability. However, this evaluator received systematic training and strictly adhered to the HAMD-17 assessment protocol throughout the evaluation process, thereby minimizing assessment bias. Future studies should increase the number of evaluators and calculate inter-rater reliability to ensure scoring consistency. Additionally, the ECT protocol in the present study was implemented based on the Chinese Expert Consensus on Modified Electroconvulsive Therapy (2019 Edition). As a result, the ECT protocol, electrode placement and treatment frequency may differ from those used in international ECT protocols. This difference constitutes one of the limitations of the present study. Future studies should meticulously document the proportion of ECT sessions without induced seizures and conduct external validation in patient cohorts treated according to international standard ECT protocols to determine the generalizability of the current findings and to identify any necessary adjustments. Finally, the study found that an improvement rate of ≥ 42.21% in HAMD-17 scores after three ECT sessions could predict the final treatment response, with high sensitivity, specificity, and PPV, but a relatively low NPV. An improvement rate of ≥ 47.05% in HAMD-17 scores after four ECT sessions could predict final remission, with high sensitivity, PPV, and NPV, but moderate specificity. It is noteworthy that in clinical decision-making, PPV and NPV may be more informative than sensitivity and specificity [38]. Therefore, early improvement rates can predict final remission with high PPV and NPV, and can also predict final response through high PPV, but the relatively low NPV should be interpreted with caution.

## Conclusion

Overall, the value of this study lies in identifying the earliest predictors of early improvement by comparing the predictive roles of improvement rates after each ECT session on final treatment outcomes. We found that an improvement rate of ≥ 42.21% in the HAMD-17 score after three ECT sessions predicted final response, whereas an improvement rate of ≥ 47.05% after four sessions predicted final remission. If early improvement signs are not achieved, it may be necessary to adjust the treatment strategy, such as modifying electrode placement or stimulus dosage. Notably, no demographic or clinical variables were found to predict ECT treatment outcomes. Future research should aim to integrate clinically relevant indicators to further explore the predictive factors influencing ECT efficacy.

## Supplementary Information

Below is the link to the electronic supplementary material.

Supplementary Material 1

## Data Availability

If required, we can provide research data with the consent of the corresponding authors.

## Abbreviations

ECT: Electroconvulsive therapy
MDD: Major depressive disorder
HAMD-17: 17-item Hamilton Depression Rating Scale
ROC: Receiver operating characteristic;
AUC: Area under the ROC curve
MADRS: Montgomery Åsberg Depression Rating Scale
DSM-V: The Diagnostic and Statistical Manual of Mental Disorders, Fifth Edition
NPV: Negative predictive value
PPV: Positive predictive value
SD: Standard deviation
CI: Confidence intervals
mITT: Modified intention-to-treat
