# Supplementary material for: Predicting the final response and remission of electroconvulsive therapy in treating major depressive disorder: exploring the earliest predictive factors for early improvement
Source: BMC Psychiatry. 2026 May 11;26:504. doi: 10.1186/s12888-026-08141-7 (PMC13330294; doi:10.1186/s12888-026-08141-7)
Supplement: Supplementary file 1 — Supplementary Material 1 [file 12888_2026_8141_MOESM1_ESM.docx]

**Table S1**

Comparison of ROC analysis and 10-fold cross-validation results.

|  | ROC cure analysis results | 10-fold cross-validation results |
| --- | --- | --- |
| Prediction of final response based on HAMD-17 improvement rate after three ECT Sessions |  |  |
| AUC | 0.838 (95% CI: 0.758-0.918) | 0.846 (95% CI: 0.744-0.949) |
| Optimal improvement rate cut-off value | 42.21% | 38.50% |
| Sensitivity | 73.64%(95% CI: 63.91%-80.94%) | 75.52% (95% CI: 66.82%-84.21%) |
| Specificity | 84.85%(95% CI: 68.25%-94.43%) | 69.20% (95% CI: 47.87%-90.65%) |
| Prediction of final remission based on HAMD-17 improvement rate after four ECT Sessions |  |  |
| AUC | 0.821 (95% CI: 0.753-0.889) | 0.822 (95% CI: 0.703-0.940) |
| Optimal improvement rate cut-off value | 47.05% | 45.71% |
| Sensitivity | 84.34% (95% CI: 74.71%-90.73%) | 84.30% (95% CI: 74.93%-93.71%) |
| Specificity | 65.00% (95% CI: 53.04%-77.23%) | 60.00% (95% CI: 38.13%-81.97%) |

Abbreviations: ROC, receiver operating characteristic; AUC, area under the receiver operating characteristic curve; CI, confidence intervals; HAMD-17, 17-item Hamilton Depression Rating Scale; ECT, electroconvulsive therapy.

**Table S2**

Demographic information and clinical characteristics of patients in the modified intention-to-treat analysis.

|  | Total | Response ^a^ | | | | Remission ^b^ | | | |
| --- | --- | --- | --- | --- | --- | --- | --- | --- | --- |
|  |  | Responders | Non-responders | Statistics | *p*-value | Remitters | Non-remitters | Statistics | *p*-value |
| Sample size | 159 | 120 | 39 | - | - | 86 | 73 | - | - |
| Age (year) | 32.27 ± 12.88 | 33.89 ± 12.75 | 27.28 ± 12.13 | *U* = 1588.000 | 0.003 ^c^ | 34.60 ± 12.58 | 29.52 ± 12.78 | *U* = 2312.000 | 0.004 ^c^ |
| Sex (male/female) | 63/96 | 48/72 | 15/24 | *χ^2^* = 0.029 | 0.865 ^d^ | 35/51 | 28/45 | *χ^2^* = 0.090 | 0.764 ^d^ |
| Education level (years) | 11.08 ± 3.75 | 10.92 ± 4.03 | 11.59 ± 2.68 | *U* = 2158.500 | 0.464 ^c^ | 10.97 ± 3.94 | 11.22 ± 3.52 | *U* = 2964.000 | 0.542 ^c^ |
| Episodes (first/recurrence) | 39/120 | 31/89 | 8/31 | *χ^2^* = 0.450 | 0.502 ^d^ | 22/64 | 17/56 | *χ^2^* = 0.112 | 0.738 ^d^ |
| Duration of illness (month) | 70.71 ± 69.76 | 75.56 ± 75.85 | 55.77 ± 43.74 | *U* = 2155.500 | 0.459 ^c^ | 76.29 ± 77.45 | 64.14 ± 59.29 | *U* = 3028.000 | 0.701 ^c^ |
| Duration of current episodes (month) | 6.28 ± 8.67 | 5.87 ± 6.95 | 7.56 ± 12.63 | *U* = 2338.000 | 0.994 ^c^ | 5.57 ± 6.47 | 7.12 ± 10.69 | *U* = 3081.000 | 0.840 ^c^ |
| Participants with  suicide attempts (yes/no) | 32/127 | 21/99 | 11/28 | *χ^2^* = 2.098 | 0.147 ^d^ | 15/71 | 17/56 | *χ^2^* = 0.839 | 0.360 ^d^ |
| Participants with Psychotic symptoms (yes/no) | 25/134 | 18/102 | 7/32 | *χ^2^* = 0.193 | 0.660 ^d^ | 13/73 | 12/61 | *χ^2^* = 0.052 | 0.819 ^d^ |
| Family history of mental illness (yes/no) | 17/142 | 11/109 | 6/33 | *-* | 0.369 ^e^ | 9/77 | 8/65 | *χ^2^* = 0.010 | 0.920 ^d^ |
| Number of ECT sessions | 7.45 ± 1.69 | 7.56 ± 1.59 | 7.13 ± 1.96 | *U* = 2097.000 | 0.279 ^c^ | 7.80 ± 1.35 | 7.04 ± 1.95 | *U* = 2563.500 | 0.027 ^c^ |
| Baseline HAMD score | 24.55 ± 6.46 | 24.28 ± 6.42 | 25.38 ± 6.58 | *t* = －0.932 | 0.353 ^f^ | 23.33 ± 6.54 | 25.99 ± 6.09 | *t* = －2.636 | 0.009 ^f^ |
| Improvement rate in total HAMD after the first ECT (%) | 23.49 ± 20.05 | 25.32 ± 20.84 | 17.87 ± 16.41 | *U* = 1733.500 | 0.015 ^c^ | 25.21 ± 22.21 | 21.46 ± 17.09 | *U* = 2728.000 | 0.155 ^c^ |
| Improvement rate in total HAMD after the second ECT (%) | 38.82 ± 23.50 | 43.19 ± 22.87 | 25.39 ± 20.33 | *U* = 1244.000 | ＜0.001 ^c^ | 44.21 ± 24.51 | 32.47 ± 20.67 | *t* = 3.231 | 0.002 ^f^ |
| Improvement rate in total HAMD after the third ECT (%) | 47.30 ± 25.34 | 54.73 ± 22.06 | 24.44 ± 20.85 | *U* = 673.000 | ＜0.001 ^c^ | 59.30 ± 21.23 | 33.17 ± 22.42 | *t* = 7.539 | ＜0.001 ^f^ |
| Improvement rate in total HAMD after the fourth ECT (%) | 53.00 ± 28.02 | 61.01 ± 24.01 | 26.46 ± 23.85 | *U* = 651.000 | ＜0.001 ^c^ | 66.60 ± 22.33 | 35.47 ± 24.76 | *U* = 995.000 | ＜0.001 ^c^ |
| Improvement rate in total HAMD after the fifth ECT (%) | 58.45 ± 28.40 | 67.71 ± 22.23 | 27.04 ± 24.42 | *U* = 381.500 | ＜0.001 ^c^ | 73.07 ± 21.85 | 38.32 ± 23.74 | *U* = 684.500 | ＜0.001 ^c^ |
| Improvement rate in total HAMD after the sixth ECT (%) | 65.04 ± 25.95 | 74.16 ± 18.35 | 34.63 ± 24.54 | *U* = 260.000 | ＜0.001 ^c^ | 79.95 ± 15.75 | 44.41 ± 23.02 | *U* = 391.000 | ＜0.001 ^c^ |

Note.

^a^ Response: 50% or more reduction in HAMD score at treatment endpoints.

^b^ Remission: HAMD score less than or equal to 7 at treatment endpoints.

^c^ Mann-Whitney *U* Test.

^d^ Pearson's *χ²* test.

^e^ Fisher's exact test.

^f^ Independent *t* test. Abbreviations: ECT, electroconvulsive therapy; HAMD, 17-item Hamilton Depression Rating Scale.

**Table S3**

**ROC analysis of clinical variables for predicting treatment response** or remission in the modified intention-to-treat analysis.

| Outcome | Clinical variables | AUC (%) | Standard error (%) | 95% CI for AUC (%) |
| --- | --- | --- | --- | --- |
| Response | Age (year) | 66.9 | 5.5 | 56.0-77.8 |
|  | Improvement rate in total HAMD after the first ECT | 61.4 | 5.2 | 51.2-71.7 |
|  | Improvement rate in total HAMD after the second ECT | 72.1 | 5.1 | 62.1-82.0 |
|  | Improvement rate in total HAMD after the third ECT | 83.8 | 4.1 | 75.8-91.8 |
|  | Improvement rate in total HAMD after the fourth ECT | 83.2 | 3.8 | 75.8-90.6 |
|  | Improvement rate in total HAMD after the fifth ECT | 89.5 | 3.4 | 82.8-96.3 |
|  | Improvement rate in total HAMD after the sixth ECT | 92.8 | 2.5 | 88.0-97.7 |
| Remission | Age | 63.0 | 4.8 | 53.5-72.5 |
|  | Number of ECT sessions | 52.4 | 4.9 | 42.8-62.0 |
|  | Baseline HAMD score | 39.7 | 4.8 | 30.4-49.1 |
|  | Improvement rate in total HAMD after the second ECT | 64.3 | 4.7 | 55.1-73.4 |
|  | Improvement rate in total HAMD after the third ECT | 79.8 | 3.8 | 72.3-87.3 |
|  | Improvement rate in total HAMD after the fourth ECT | 82.1 | 3.4 | 75.3-88.9 |
|  | Improvement rate in total HAMD after the fifth ECT | 86.8 | 3.0 | 80.8-92.8 |
|  | Improvement rate in total HAMD after the sixth ECT | 92.1 | 2.2 | 87.8-96.5 |

Abbreviations: ROC, receiver operating characteristic; AUC, area under the receiver operating characteristic curve; CI, confidence intervals; HAMD, 17-item Hamilton Depression Rating Scale; ECT, electroconvulsive therapy.

**Table S4**

Predictive performance of early HAMD-17 Improvement in the modified intention-to-treat analysis.

| Outcome | Optimal cut-off values for improvement | Sensitivity  (true positive) | Specificity  (true negative) | False positive  (100% －specificity) | False negative  (100% －sensitivity) | PPV | NPV |
| --- | --- | --- | --- | --- | --- | --- | --- |
| Response | 42.21% | 74.17% | 87.18% | 12.82% | 57.79% | 94.68% | 52.31% |
| Remission | 47.06% | 84.71% | 62.12% | 37.88% | 15.29% | 74.23% | 75.93% |

Abbreviations: PPV, positive predictive value; NPV, negative predictive value; HAMD-17, 17-item Hamilton Depression Rating Scale; ECT, electroconvulsive therapy.
